# Patterns of sexual violence in Eastern Democratic Republic of Congo: reports from survivors presenting to Panzi Hospital in 2006

**DOI:** 10.1186/1752-1505-4-9

**Published:** 2010-05-05

**Authors:** Susan A Bartels, Jennifer A Scott, Denis Mukwege, Robert I Lipton, Michael J VanRooyen, Jennifer Leaning

**Affiliations:** 1 Department of Emergency Medicine, Beth Israel Deaconess Medical Center, Boston, USA; 2 Harvard Humanitarian Initiative, Cambridge, USA; 3 Department of Obstetrics and Gynecology, Beth Israel Deaconess Medical Center, Boston, USA; 4 Hôpital de Panzi, Bukavu, South Kivu, Democratic Republic of Congo; 5 Department of Emergency Medicine, Brigham and Women's Hospital, Boston, USA; 6 Harvard School of Public Health, Boston, USA

## Abstract

**Background:**

Despite the signing of international peace agreements, a deadly war continues in the Democratic Republic of Congo (DRC) and sexual violence is a prominent modus operandi of many military groups operating in the region.

**Methods:**

Retrospective cohort study of women who presented to Panzi Hospital in 2006 requesting post-sexual violence care. Data was extracted and analyzed to describe the patterns of sexual violence.

**Results:**

A total of 1,021 medical records were reviewed. A majority of attacks occurred in individual homes (56.5%), with the fields (18.4%) and the forest (14.3%) also being frequent locations of attack. In total, 58.9% of all attacks occurred at night. Of the four primary types of sexual violence, gang rape predominated (59.3%) and rape Not Otherwise Specified (NOS) was also common (21.5%). Sexual slavery was described by 4.9% of the survivors and a combination of gang rape and sexual slavery was described by 11.7%. The mean number of assailants per attack was 2.5 with a range of one to > 15. There were several demographic predictors for sexual slavery. Controlling for age, education level and occupation, a marital status of "single" increased the risk of sexual slavery (OR = 2.97, 95% CI = 1.12-7.85). Similarly, after controlling for other variables, age was a significant predictor of sexual slavery with older women being at a slightly reduced risk (OR = 0.96, 95% CI = 0.92-0.99). Women who experienced sexual slavery were 37 times more likely to have a resultant pregnancy in comparison to those who reported other types of sexual violence (OR = 37.50, 95% CI = 14.57-99.33).

**Conclusions:**

Among sexual violence survivors presenting to Panzi Hospital in 2006, the majority of attacks occurred in women's own homes, often at night. This represents a pattern of violence that differs from other conflict settings and has important implications regarding protection strategies. Sexual violence in South Kivu was also marked with a predominance of gang rape, thus increasing the risk of serious injury as well as the likelihood of an individual woman contracting a sexually transmitted infection (STI). Sexual slavery was noted to be more common among young, single women and was found to have a high rate of resultant pregnancy.

## Background

Sexual violence in the Democratic Republic of the Congo (DRC) has been one of the most devastating aspects of the armed conflict that began in 1996 [1-8]. Sexual violence has been employed by militia groups to intimidate and punish communities and to control territory. Rape and sexual torture have systematically destroyed communities and the dignity of its survivors[9], and has been an impediment to achieving peace and stability within the region.

After the Rwandan civil war and genocide in 1994, a massive influx of refugees from Rwanda flowed into Eastern Zaire (now the DRC). In addition to refugees, several militia groups entered the region. The ensuing civil conflicts of 1996 and 1998 have led to a protracted state of violence, claiming over five million lives since 1996[10]. Despite the promise of peace accords in 2003 and 2008, as well as the nation's first free elections in 2006, militia activity and lawlessness persists, especially in the eastern provinces.

While sexual violence has been a known feature of armed conflict throughout history, the scale and extent of the violence in Eastern DRC is unprecedented in modern times. The practice is widespread in the provinces of North and South Kivu. Many of the armed groups operating in North and South Kivu have participated in sexual atrocities, including the the National Congress for the Defence of the People (CNDP), the Democratic Forces for the Liberation of Rwanda (FDLR) and local Congolese militias. The United Nations (UN) Security Council has condemned all forms of sexual violence during armed conflict as weapons of war and has called for an immediate and complete halt to these acts[11].

The incidence of sexual violence has been challenging to accurately estimate due to stigmatization associated with the violence, due to the vulnerability of women who speak out or seek medical care and due to the general insecurity in Eastern DRC. In 2008 the International Rescue Committee reported having assisted over 40,000 rape survivors in DRC since 2003, and Médecins Sans Frontières (MSF) reports treating an average of 60 survivors per month[5]. Furthermore, the UN reports 27,000 sexual assaults in South Kivu Province alone in 2006[5].

Panzi Hospital in Bukavu, the capital of South Kivu Province, serves as a major general referral hospital offering services in obstetrics/gynecology, pediatrics, internal medicine, surgery, dentistry and nutrition. Established in 1999, the 334-bed hospital receives approximately 10 sexual violence survivors daily[12] under the center's *Victims of Sexual Violence Program*. The *Victims of Sexual Violence Program *provides sexual violence survivors with free medical treatment and free psychological and spiritual care in addition to socio-economic assistance.

There are many unanswered questions regarding the sexual violence epidemic in Eastern DRC. For example, what are the patterns of sexual violence and how might an understanding of these patterns be used to protect Congolese women? What is the actual extent of sexual violence in Eastern DRC? What are the physical and psychosocial consequences of sexual violence in this context and how might resources be better allocated to help survivors? To help address these questions, researchers from the Harvard Humanitarian Initiative, in collaboration with Panzi Hospital staff, performed a retrospective cohort study of sexual violence survivors presenting to Panzi Hospital in 2006. Specifically, this study focuses on the patterns of sexual violence with particular reference to: 1) characteristics of attacks including location, time of day, circumstances and alleged perpetrators; 2) demographic predictors of types of sexual violence; and 3) physical, psychological and social consequences according to the type of sexual violence experienced.

## Methods

This is a retrospective cohort study conducted at Panzi Hospital. Using a non-systematic convenience sample, interviews were conducted with sexual violence survivors as they presented to hospital during 2006. Individual women were chosen for interview based on staff availability and the perceived severity of physical/psychological trauma at time of triage. The interviews were conducted in private by trained female nurses using a two-paged, semi-structured questionnaire. The interviews were conducted in French or in the local dialect (Kiswahili or Mashi) and the information was documented in French. The questionnaire asked basic demographic information and then allowed the woman to describe her sexual violence experience and subsequent consequences in an open, self-reporting format. All data sheets were subsequently filed in a locked administrative office at Panzi Hospital and were kept independent of the patient's hospital record.

Between November 2007 and March 2008, a total 1,021 records were reviewed from sexual violence survivors who received care in 2006 under Panzi Hospital's *Victims of Sexual Violence Program*. In the same period, another 1,851 women accessed post-sexual violence care at Panzi Hospital. However, because of staffing limitations these women did not undergo the in-depth interview and detailed information on their sexual assaults was not captured in this study.

For each questionnaire a single sexual violence experience was recorded. It was rare that more than one sexual assault was described in a single questionnaire and in the instances when there was more than one sexual assault described, the most recent experience of sexual violence was recorded for study purposes. Thus, there are 1021 sexual violence experiences in this dataset.

Data were extracted from interview records and entered into an electronic spreadsheet (Excel Version 11.5.6). Analysis was performed using SAS Version 9.2 (The SAS Institute). This study was approved by the Institutional Review Board at the Harvard School of Public Health and by the medical director of Panzi Hospital.

For the purposes of this study, "sexual violence" was defined as any unwanted physical contact of a sexual nature. It included forced vaginal or anal intercourse, forced oral sex, penetration with a foreign object, forced sexual acts betweens victims, being forced to undress and sexual harassment. "Gang rape" was defined as sexual violence committed by two or more assailants. "Sexual slavery" was defined as being held captive for the purpose of sexual violence for more than 24 hours. "Rape Not Otherwise Specified" (NOS) was taken to be sexual violence committed by a single assailant and not involving sexual slavery. It was also used to describe sexual violence in which the survivor simply stated that she was raped without providing any further details.

## Results

In 2006, a total of 1,851 survivors presented to Panzi Hospital requesting care under the *Victims of Sexual Violence Program*. Resources permitted detailed interviews to be conducted with 1,021 of these women. Table 1 outlines the population demographics disaggregated by type of violence. For the purposes of this analysis, there were four major categories of sexual violence: 1) rape NOS; 2) gang rape; 3) sexual slavery; and 4) a combination of gang rape and sexual slavery. Twenty-eight survivors (2.7%) reported other types of sexual violence including forced oral sex, anal intercourse, forced rape between victims, penetration with a foreign object, being forced to undress and sexual harassment. Because the number of incidents in each of these categories was small, these sexual violence experiences have not been included in the analysis.

**Table 1:** Demographics of survivors presenting to Panzi Hospital in 2006 according to type of sexual violence experienced.


	**Rape NOS**		**Gang Rape**		**Sexual Slavery**		**Combined Gang Rape and Sexual Slavery**	

	**Number**	**Percent**	**Number**	**Percent**	**Number**	**Percent**	**Number**	**Percent**

**Age**								
≤15	35	16.0	50	8.3	12	24.0	11	9.2
16 - 35	81	37.0	250	41.3	28	56.0	78	65.6
36 - 55	83	37.9	264	43.6	10	20.0	29	24.4
≥ 55	20	9.1	41	6.8	0	0	1	0.84
Total	219	100	605	100	50	100	119	100

**Marital Status**								
Single	41	18.7	43	7.1	21	42.0	17	14.3
Married	100	45.7	308	50.9	18	36.0	50	42.0
Widowed	46	21.0	148	24.5	3	6.0	28	23.5
Abandoned	32	14.6	102	16.9	7	14.0	24	20.2
Not Specified	0	0	4	0.67	1	2.0	0	0
Total	219	100	605	100	50	100	119	100

**Education**								
Illiterate	147	67.1	427	70.6	21	42.0	63	52.9
Primary School	52	23.7	138	22.8	19	38.0	44	37.0
Secondary School	16	7.3	38	6.3	9	18.0	11	9.2
Post-Secondary	1	0.46	0	0	0	0	0	0
Not Specified	3	1.4	2	0.33	1	2.0	1	0.8
Total	219	100	605	100	50	100	119	100

**Occupation**								
Farmer	167	76.3	483	79.8	31	62.0	86	72.3
Student	19	8.7	53	8.8	8	16.0	18	15.1
Trader	17	7.7	18	3.0	7	14.0	4	3.4
Laborer	10	4.6	32	5.3	1	2.0	7	5.9
Teacher	0	0	7	1.2	0	0	0	0
Unemployed	0	0	2	0.33	0	0	0	0
Not Specified	2	0.91	8	1.3	2	4.0	3	2.5
Other	4	1.8	2	0.33	1	2.0	1	0.84
Total	219	100	605	100	50	100	119	100

**Ethnicity**								
Bashi	142	64.8	372	61.5	29	58.0	80	67.2
Barega	18	8.2	66	10.9	9	18.0	7	5.9
Bahavu	9	4.1	24	4.0	0	0	6	5.0
Bafulero	15	6.9	45	7.4	0	0	3	2.5
Batembo	21	9.6	54	8.9	8	16.0	14	11.8
Not Specified	4	1.8	2	0.33	2	4.0	1	0.84
Other	10	4.6	42	6.9	2	4.0	8	6.7
Total	219	100	605	100	50	100	119	100

### Attack Characteristics

Each rape experience had only one code for perpetrator even though there may have been a group of perpetrators (example: a group of *Mai Mai *perpetrators would have had a single perpetrator code of *"Mai Mai"*). Additionally, there were no mixed perpetrators described in this dataset (example: a civilian perpetrator and a military perpetrator implicated in the same rape or mixed armed groups implicated in the same rape), thus each perpetrator code is exclusive.

The mean number of assailants per sexual assault was 2.5 with a median of 2 and a range of 1 to greater than 15 assailants. In total, 512 women (50.0%) described the perpetrator(s) as "assailant(s)" and no further identifying information could be gathered. For the purposes of this analysis, these perpetrators are simply referred to as "not specified". Another 280 women (27.3%) described the assailant(s) as being either "soldier(s)" or "man/men in military uniform" without mention of a particular military affiliation. These perpetrators are referred to as "soldiers Not Otherwise Specified" ("soldiers NOS") throughout this analysis. Two hundred and ten women (20.5%) identified their perpetrators as belonging to a specific military group and these perpetrators are referred to as "named soldiers". Specific military affiliations included *Interahamwe*, Hutu soldiers, FARDC, *Mai Mai*, Nkunda soldiers, Congolese soldiers, Tutsi soldiers, *Soldats de 106*, Rwandan soldiers, FDD, RCD, *Mudundu 40*, *Mutebutsi*, and *Rasta*. The remaining 23 women (2.2%) described their assailants as being civilian.

The majority of attacks occurred in the woman's own home (577 or 56.5%) with another 146 taking place in the forest (14.3%), 188 taking place in the fields (18.4%) and 68 taking place while the woman was walking or traveling along a road (6.7%). The final 4.1% of attacks occurred in other locations including the market, water sources, public buildings, or someone else's home (often the perpetrator's).

In total, 601 or 58.9% of the attacks occurred at night, 349 occurred during the day (34.2%) and 57 (5.6%) occurred during evening hour. The remaining 1.3% of women did not specify the time of day they were attacked.

A majority of the sexual assaults were gang rape (1,021 or 59.3%). Rape NOS accounted for 219 of the attacks (21.5%) and there were 50 accounts of sexual slavery (4.9%). Another 119 women described a combination of gang rape and sexual slavery (11.7%) and the remaining 28 women (2.7%) described other types of sexual violence as outlined above.

When the characteristics of attack were disaggregated according to type of sexual violence several important patterns emerged (Table 2). First, women who were attacked in the forest were twice as likely to describe a combination of gang rape and sexual slavery than were women who were attacked in other locations (Odds Ratio [OR] = 2.13, 95% Confidence Interval [CI] = 1.30 - 3.46). Women who were attacked in the fields were more likely to experience gang rape than were women who were attacked in other locations (OR = 1.58, 95% CI = 1.10 - 2.26). Sexual violence survivors who were attacked during the night were almost two and a half times more likely to describe a combination of gang rape and sexual slavery (OR = 2.44, 95% CI = 1.54 - 3.91) than were women who were attacked during the day.

**Table 2:** Patterns of attack among survivors presenting to Panzi Hospital in 2006 according to type of sexual violence experienced.


	**Rape NOS**		**Gang Rape**		**Sexual Slavery**		**Combined Gang Rape and Sexual Slavery**	

	**Number**	**Percent**	**Number**	**Percent**	**Number**	**Percent**	**Number**	**Percent**

**Location**								
Own Home	117	53.4	356	58.8	24	48.0	68	57.1
Forest	31	14.2	72	11.9	12	24.0	29	24.4
Fields	40	18.2	127	21.0	6	12.0	10	8.4
Roads	21	9.6	35	5.8	4	8.0	6	5.0
Other	3	1.4	5	0.83	0	0	0	0
Not Specified	7	3.2	10	1.65	4	8.0	6	5.0
Total	219	100	605	100	50	100	119	100

**Time**								
Day	80	36.5	216	35.7	20	40.0	24	20.2
Evening	21	9.6	26	4.3	4	8.0	4	3.4
Night	114	52.1	359	59.3	26	52.0	91	76.5
Not Specified	4	1.8	4	0.66	0	0	0	0
Total	219	100	605	100	50	100	119	100

**Circumstances**								
Sleeping	67	30.6	273	45.1	22	44.0	69	58.0
Working	46	21.0	133	22.0	4	8.0	14	11.8
Traveling	16	7.3	38	6.3	5	10.0	9	7.6
Going to/from market	17	7.7	23	3.8	4	8.0	1	0.84
Relaxing	22	10.1	36	5.9	3	6.0	5	4.2
Hiding	6	2.7	30	4.9	2	4.0	7	5.9
Not Specified	13	5.9	23	3.8	3	6.0	6	5.0
Other	32	14.6	49	8.1	7	14.0	8	6.7
Total	219	100	605	100	50	100	119	100

**Perpetrator**								
Soldier NOS	66	30.1	168	27.7	16	32.0	22	18.5
Named Soldier	30	13.7	116	19.1	13	26.0	43	36.1
Civilian	12	5.5	7	1.2	1	2.0	0	0
Not Specified	111	50.7	314	52.0	20	40.00	54	45.4
Total	219	100	605	100	50	100	119	100

The type of perpetrator was also predictive of the type of sexual violence. For instance, women who were attacked by civilian perpetrators were five times more likely to experience rape NOS as compared to women who were attacked by other types of perpetrators (OR = 5.55, 95% CI = 2.08 - 15.06). Furthermore, women who were attacked by named soldiers were more likely to describe a combination of gang rape and sexual slavery than were women who were attacked by other types of perpetrators (OR = 2.54, 95% CI = 1.65 - 3.92).

### Demographic Predictors of Type of Sexual Violence

Demographic differences in patterns of sexual slavery were notable. For instance, women who were 35 years of age or younger were more likely to be taken as sex slaves in comparison with women who were over the age of 35 (OR = 3.47, 95% CI = 1.65 - 7.49). Women and girls who were single, without ever having been married, were six times more likely to be taken as sex slaves in comparison to women who were married, abandoned, or widowed (OR = 6.04, 95% CI = 3.18 - 11.42). Women reporting sexual slavery were also more likely to be educated with those having attended secondary school being almost three times more likely to be taken as sex slaves in comparison to women who reported being illiterate or reported having attended only primary school (OR= 2.97, 95% CI = 1.28 - 6.68). With regards to occupation, women who were taken as sex slaves were more likely to report being students (OR = 4.13, 95% CI = 2.62 - 6.46) and more likely to report being traders (OR = 3.77, 95% CI = 1.45 - 9.44). Using logistic regression modeling to control for age, education level and occupation, a marital status of "single" still placed women at significant risk for sexual slavery (OR = 2.97, 95% CI = 1.12 - 7.85). Similarly, age was still a significant predictor of sexual slavery when logistic regression modeling controlled for marital status, education and occupation, with older women being at a slightly reduced risk of sexual slavery (OR = 0.96, 95% CI = 0.92 - 0.99).

Other significant demographic predictors of type of sexual violence were identified. Women who were 16 to 35 years of age were more likely to experience a combination of gang rape and sexual slavery than were women falling into other age categories (OR = 2.73, 95% CI = 1.79 - 4.16). Also, women who belonged to the Barega tribe were three times more likely to report "other" types of sexual violence in comparison to women who self-identified with other ethnicities (OR = 2.98, 95% CI = 1.12 - 7.62). The "other" types of sexual violence included forced oral sex, anal intercourse, forced rape between victims, penetration with a foreign object, being forced to undress and sexual harassment.

### Consequences

The reported consequences of sexual assault are outlined in Table 3. One of the most devastating consequences of sexual violence was resultant pregnancy, which was reported by a total of 62 women in this study (6.1%). Pregnancy was reported by 12 women experiencing rape NOS (5.5%), by 13 women experiencing gang rape (2.1%), by 20 women experiencing sexual slavery (40.0%) and by 17 women experiencing combined gang rape and sexual slavery (14.3%). The type of sexual violence was a significant predictor of this outcome. Pregnancy was 37 times more common following sexual slavery than it was following other types of sexual violence (OR = 37.50, 95% CI - 14.57 - 99.33). Pregnancy was also more common among women who experienced a combination of gang rape and sexual slavery although not to the same degree (OR = 2.64, 95% CI = 1.38 - 5.01). In contrast, women who experienced gang rape were much less likely to report a resultant pregnancy (OR = 0.15, 95% CI = 0.08 - 0.29). The type of sexual violence was also predictive of reporting pain. Women who experienced sexual slavery were significantly less likely to report pelvic, lumbar and abdominal pain than were women who experienced other types of sexual violence (OR = 0.12, 95% CI = 0.03 - 0.35).

**Table 3:** Physical, social and psychological consequences reported by survivors presenting to Panzi Hospital in 2006 according to type of sexual violence experienced.

	Rape NOS		Gang Rape		Sexual Slavery		Combined Gang Rape and Sexual Slavery	
	**Number**	**Percent**	**Number**	**Percent**	**Number**	**Percent**	**Number**	**Percent**

**Physical:**								
Pelvic pain	53	24.2	139	23.0	3	6.0	25	21.0
Lumbar pain	24	11.0	72	11.9	0	0	16	13.4
Abdominal pain	22	10.0	41	6.8	1	2.0	6	5.0
Pregnancy	12	5.5	13	2.1	20	40.0	17	14.3
Malaise	10	4.6	44	7.3	1	2.0	6	5.0
Vaginal discharge	10	4.6	36	6.0	1	2.0	8	6.7
Vaginal itching	9	4.1	25	4.1	1	2.0	8	6.7
Abnormal menses	10	4.6	26	4.3	1	2.0	6	5.0
Generally unwell	5	2.3	24	4.0	0	0	5	4.2

**Social:**								
Loss of possessions	55	25.1	147	24.3	10	20.0	19	16.0
Loss of child/spouse	13	5.9	75	12.4	2	4.0	21	17.6
Spousal abandonment	6	2.7	43	7.1	3	6.0	10	8.4
Loss of other family	2	0.91	15	2.5	0	0	9	7.6
Loss of home	1	0.46	9	1.5	0	0	1	0.84

**Psychological:**								
Anxiety about rape	53	24.2	154	25.5	18	36	31	26.1
Anxiety about spousal abandonment	6	2.7	38	6.3	4	8	9	7.6
Concern about STIs	25	11.4	76	12.6	4	8	19	16.0
Concern about HIV	14	6.4	56	9.3	4	8	13	10.9
General health concerns	16	7.3	66	10.9	7	14	9	7.6
Grief about loss of spouse	9	4.1	40	6.6	1	2	10	8.4
Grief about loss of child/children	8	3.7	25	4.1	0	0	3	2.5

Women could report more than one consequence for each of the three categories. Due to the self-reporting format of the interview, these figures undoubtedly underestimate specific outcomes since failure to mention a specific symptom or outcome does not imply its absence.

Social consequences of sexual violence often involved the death of children, death of spouses, spousal abandonment, death of other family members, loss of possessions (such as cash, food, clothing, and livestock) and occasionally, loss of the family home. In some instances the risk of these outcomes depended on the type of sexual violence experienced. Women who described a combination of gang rape and sexual slavery were almost four times more likely to report death of other family members (9/119 or 7.6%) in comparison to women who experienced other types of sexual violence (OR = 3.78, 95% CI = 1.47 - 9.57). Loss of a child/spouse was more commonly reported by survivors of gang rape (75/605 or 12.4%) and by survivors describing a combination of gang rape and sexual slavery (21/119 or 17.6%) (OR = 1.95, 95% CI = 1.03 - 3.72).

Using the above four categories of sexual violence, psychological consequences did not differ significantly according to type of violence.

## Discussion

Analysis of this dataset, consisting of 1,021 survivors presenting to Panzi Hospital seeking post-sexual violence care in 2006, illustrated that sexual violence is pervasive in South Kivu. It affects young, old, single, married, divorced, and widowed women. It affects the educated and the uneducated, as well as women of different occupations and different ethnicities. In general, the educational statuses [13,14], occupations[15], and ethnicities[14] of women in this dataset are believed to reflect the general demographics of women in South Kivu Province.

One of the most important patterns identified in this analysis is that the majority of sexual assaults occur in individual homes. Individual homes were the most commonly reported location regardless of whether the sexual violence consisted of rape NOS, gang rape, sexual slavery or a combination of gang rape and sexual slavery (with sexual slavery, the attack was initiated in, and the woman was captured, from her own home). This finding contrasts with sexual violence in other conflicts where women often become victims to sexual violence outside the home. For instance, MSF reported that in Darfur, 82% of sexual assaults were initiated while women were outside their home villages, usually in search of firewood, collecting water, or farming their fields[16].

Furthermore, this analysis revealed that most sexual assaults occurred at night, often while women were sleeping next to their spouses or families. A majority of attacks happening at night corresponds with the above finding that a majority of attacks occur in individual homes since most women in South Kivu Province are farmers, spending their days working in the fields and returning home at night to be with their families. Unfortunately, spousal presence at the time of attack or the presence of other male family members did little to deter the sexual violence perpetrator(s). Survivors often reported that spouses and male family members were severely beaten, restrained, killed or forced to witness the sexual violence.

This predominant pattern of attack, on individual homes at night, has important implications for the protection of women in South Kivu. Protection of women and girls during conflict has become a greater priority since the United Nations (UN) Security Council passed Resolution 1820 in 2008, recognizing that sexual violence is commonly used as a strategic weapon of war and calling for a response to the problem[11]. In conflict settings other than DRC, that response has included interventions such as the provision of fuel-efficient stoves to reduce the frequency with which women have to leave the village in search of firewood[17]. Other responses have included armed firewood patrols that accompany women on their long treks to collect firewood or water[17]. In Eastern DRC, however, such measures are likely to be met with limited success, since at least in the current dataset, a majority of attacks happen at night in individual homes. Data on the location and circumstances of sexual assaults are critical for the development and implementation of successful protection programs. By strategizing with local community members, the UN and other aid organizations should aim to identify new and innovative protection programs based on the patterns of attack that are now being recognized to be most prevalent in South Kivu.

In total, 71% of women presenting to Panzi Hospital in 2006 had experienced gang rape (59.3% reported gang rape and 11.7% reported a combination of gang rape and sexual slavery). The mean number of assailants per sexual assault was 2.5 and a few women were assaulted by more than 15 perpetrators. From a medical standpoint, this preponderance of gang rape has important health implications. Each assault by a different perpetrator increases the likelihood that the woman will contract a Sexually Transmitted Infection (STI) including Human Immunodeficiency Virus (HIV). Women who are assaulted repeatedly by different perpetrators may also be at higher risk for genital trauma and bodily injury. An individual survivor's intake questionnaire was not linked to her hospital medical record, thus the prevalence of STIs and physical injury could not be assessed in this analysis. The psychological consequences of sexual violence also appear to be partially dependent on the number of assailants. Prior work at Panzi Hospital illustrated that women reporting multiple assailants were more likely to report psychological stress than were women who reported being assaulted by a single perpetrator[18].

That gang rape was so prevalent in this dataset speaks to the widespread acceptance of sexual violence and violence against women in South Kivu Province. For those gang rapes committed by military perpetrators, the high prevalence of gang rape suggests that sexual violence may be used as a method of male bonding and may be offered to combatants as a reward for bravery or victory. Gang rape by military perpetrators also implies that military leaders either support or condone such behavior despite the fact that sexual violence is a war crime according to the Fourth Geneva Convention[19] and a crime against humanity according to the Rome Statue of the International Criminal Court[20]. Alternatively, it could imply that the involved militias are so undisciplined and the command structure so weak, that military leaders exert little or no authority over their soldiers. Gang rape committed by non-military perpetrators suggests a widespread acceptance of sexual violence among local Congolese society and speaks to the environment of impunity that continues to exist in Eastern DRC.

Disaggregation of this data according to type of sexual violence revealed important patterns of attack. One of the most salient patterns was that young, unmarried women were more likely to be taken into sexual slavery. Sexual violence for any woman and for any generation of women is a devastating and horrific criminal act. However, it is perhaps more disruptive to society as a whole for young, educated women to be specifically affected. It is this generation of Congolese women who have the opportunity to become breadwinners for their families, the opportunity to become leaders within their professions and through their success, the opportunity to advance the overall status of women in DRC. Thus, for these young women to have their health, both physical and psychological, compromised by an experience of sexual slavery, could be particularly harmful to the status of Congolese women and to Congolese society in general.

Survivors who experienced sexual slavery were 37 times more likely to become pregnant as a result of the violence. Previous work from Panzi Hospital demonstrated that sexual violence survivors who become pregnant as a result of the attack were more likely to report psychological symptoms than were women who did not report a resultant pregnancy[18]. Since abortion is illegal in DRC and since adoption is rarely considered an option in Congolese society, most pregnant sexual violence survivors find themselves responsible for raising a child. Because many sexual slavery survivors are single and because identification as a "rape victim" usually impedes a woman's chances of marrying, many pregnant survivors are single parents. Not only does this impede the survivor's ability to continue school or to pursue a career, but without the economic support and protection traditionally provided by men in DRC, it also increases her vulnerability.

This study has several limitations. First, because it is retrospective in nature the original information and the manner in which it was collected cannot be verified or validated. The retrospective nature also prevents clarification of documentation inconsistencies and has resulted in missing data. Furthermore, it is impossible to make causal claims from the data. For instance, a sexual violence victim with specific symptoms may have had these symptoms before the sexual violence.

Second, this study was limited by its sampling methodology. Because the data are representative only of those sexual violence survivors presenting to Panzi Hospital for post-sexual violence care, there is an inherent selection bias. Despite this inherent selection bias, the data still allow for a deeply descriptive understanding of women in this setting. The sampling within Panzi Hospital was also a limitation. The *Victims of Sexual Violence Program *was sporadically understaffed, meaning that at times there were an insufficient number of trained staff to conduct all the necessary interviews. During these times of understaffing, the existing intake staff chose for interview those women whom they believed to have suffered the most traumatic violence, based on interactions during the initial triage. The data registry gaps appeared to have arisen sporadically as a result of insufficient staffing. It is possible that the 1,021 sexual violence survivors presented here do in fact represent the more extreme cases on the spectrum of violence, since they were apparently selected on that basis for interview by the staff. However, we believe that it would be challenging to determine at first glance during the registration process which women had suffered the most severe trauma.

A third limitation of this study was the open, self-reporting format. Without asking specific questions regarding consequences of rape, our study undoubtedly underestimates specific outcomes. Failure to mention a specific symptom or outcome does not imply its absence.

And finally, several translations were required before the analysis of these data (Kiswahili or Mashi to French, French to English), thus introducing the potential for error. Additionally, cultural differences have the potential to introduce error into the analysis. To limit this potential source of error, the results were discussed with local staff at Panzi Hospital who provided cultural background and context.

Future work will address several of these limitations. Next steps include the implementation of a prospective sexual violence registry at Panzi Hospital to include all women requesting post-sexual violence care. This registry will be developed using a revised questionnaire with specific questions on the location, time and circumstances of the attack as well as the number and identification of perpetrators and the type of sexual violence. Direct questions will also be asked to better define the physical, psychological and social consequences of being raped. It will also investigate traumatic fistulas, which this study was not designed to address.

## Conclusions

In South Kivu Province, sexual violence affects women of all ages and from all different ethnicities regardless of marital status, education level or occupation. Within this dataset, a majority of the attacks occur in the women's own homes, often at night and in the presence of their family members. This pattern of sexual violence differs from that which has been reported in other conflict areas and has important implications regarding protection strategies. The UN and other humanitarian organizations implementing protection programs in Eastern DRC should consider strategizing with local women and community leaders to devise new protection protocols specific to the attack patterns within the region. Sexual violence in South Kivu was also marked with a predominance of gang rape, thus increasing the risk of serious injury as well as the likelihood of contracting an STI. The high prevalence of gang rape implies a widespread acceptance of sexual violence both within military groups and also within local Congolese society. Sexual slavery was noted to be more common among young, single women and was found to have a high rate of resultant pregnancy. That the next generation of young Congolese women is being specifically affected by sexual slavery and its resultant pregnancies, may have important economic and societal implications since the experience likely challenges the ability of these women to become professional and community leaders, and thus limits their opportunity to advance the status of women in Eastern DRC.

## Abbreviations

CI: Confidence Interval; DRC: Democratic Republic of Congo; FARDC: Forces Armées de la République Démocratique du Congo; FDD: Forces pour la Défense de la Démocratie; HIV: Human Immunodeficiency Virus; MSF: Médecins sans Frontières; NOS: Not Otherwise Specified; OR: Odds Ratio; RCD: Rassemblement Congolais pour la Démocratie; STI: Sexually Transmitted Infection; UN: United Nations

## Competing interests

The authors declare that they have no competing interests.

## Authors' contributions

SB extracted, entered and helped to analyze the data. SB also aided in preparing the manuscript. JS extracted and entered the data and helped with writing the manuscript. JL guided analysis of the data and helped write the manuscript. DM provided help with extracting and entering the data, provided important context and background for the discussion and critiqued the manuscript. RL performed most of the analysis and reviewed the manuscript. MV helped design the study, guided analysis and helped prepare the manuscript.

All authors have read and approved this final manuscript.
